# RNF213 and GUCY1A3 in Moyamoya Disease: Key Regulators of Metabolism, Inflammation, and Vascular Stability

**DOI:** 10.3389/fneur.2021.687088

**Published:** 2021-07-26

**Authors:** Yohei Mineharu, Susumu Miyamoto

**Affiliations:** Department of Neurosurgery, Kyoto University Graduate School of Medicine, Kyoto, Japan

**Keywords:** moyamoya disease, RNF213 mutation, GUCY1A3, CAV1 (caveolin-1), calcineurein/NFAT pathway, nitric oxide, inflammation, vascular

## Abstract

Moyamoya disease is an idiopathic chronically progressive cerebrovascular disease, which causes both ischemic and hemorrhagic stroke. Genetic studies identified *RNF213*/*Mysterin* and *GUCY1A3* as disease-causing genes. They were also known to be associated with non-moyamoya intracranial large artery disease, coronary artery disease and pulmonary artery hypertension. This review focused on these two molecules and their strong linker, calcineurin/NFAT signaling and caveolin to understand the pathophysiology of moyamoya disease and related vascular diseases. They are important regulators of lipid metabolism especially lipotoxicity, NF-κB mediated inflammation, and nitric oxide-mediated vascular protection. Although intimal thickening with fibrosis and damaged vascular smooth muscle cells are the distinguishing features of moyamoya disease, origin of the fibrous tissue and the mechanism of smooth muscle cell damages remains not fully elucidated. Endothelial cells and smooth muscle cells have long been a focus of interest, but other vascular components such as immune cells and extracellular matrix also need to be investigated in future studies. Molecular research on moyamoya disease would give us a clue to understand the mechanism preserving vascular stability.

## Introduction

Moyamoya disease (MMD) is a progressive occlusive cerebrovascular disease of unknown etiology (1). Main treatment strategy is revascularization surgery to prevent cerebrovascular events, and antiplatelet therapy is another treatment option that has recently been reappraised (2, 3). However, there is no curative treatment that can prevent the progression of arterial stenosis. Understanding the molecular biology of MMD is needed to develop a new treatment strategy and proper biomarkers to evaluate therapeutic efficacy.

Genetics has contributed greatly to understanding the pathophysiology of MMD. In 2011, *RNF213* (also called as *Mysterin*) was identified as the major susceptibility gene for MMD and p.R4810K mutation was found as a founder mutation that increases the risk of MMD by ~300 times (4). The mutation was detected in ~80% of patients with MMD in Japan, ~70% in Korea and ~30% in China. Although the same mutation was not detected in European patients, other mutations such as p.D4013N were found in ~10% (5). Thus, *RNF213* is a major susceptibility gene for MMD and it has been recognized as a key molecule to understand the pathophysiology of MMD.

It has been reported that patients with MMD have not only cerebrovascular lesions but also extracranial lesions including coronary artery disease and pulmonary artery stenosis. Importantly, these lesions have common pathological features (6). In agreement with this finding, the p.R4810K mutation in *RNF213* gene was also shown to be associated with coronary artery disease (7, 8), pulmonary artery hypertension (9), and renal artery stenosis (10). *GUCY1A3* mutations were first detected in patients with quasi-MMD (syndromic MMD) with achalasia (11), but some cases show only moyamoya arteriopathy in the absence of achalasia (12). Of note, *GUCY1A3* is also known to be associated with coronary artery disease (13) and pulmonary artery hypertension (14) as is the case with *RNF213*. Both *RNF213* and *GUCY1A3* are also associated with hypertension. These lines of evidence suggest that investigation of these two genes may give us some clues to elucidate the molecular pathogenesis of MMD and quasi-MMD.

In this narrative review, we will discuss characteristics of *RNF213* and *GUCY1A3* in terms of (1) genetics, (2) molecular structures and functions, (3) transcriptional regulation, (4) possible roles in vascular wall, (5) possible roles in vascular insults, and (6) other properties. Finally, we will propose a potential mechanism of MMD and future research directions.

## Genetics of *RNF213* and *GUCY1A3*

Susceptibility locus for MMD have been reported to reside on 3p, 8q23, and 17q25.3. Chromosome 17q locus was first identified by chromosome wide linkage analysis (15), and it was narrow down to the 3.5-Mb region at 17q25.3 (16) by linkage analysis of families with autosomal dominant inheritance with incomplete penetrance. In this locus, *RNF213* was identified as the major susceptibility gene for MMD (4). In East Asian patients, the p.R4810K mutation is most common, and it is also found in 2% of the general population, which is in agreement with low penetrance (1/100–1/200) of the gene. Intrudingly, genotypes of *RNF213* have impact on clinical features of MMD. The p.R4810K mutation was associated with severer form of the disease with higher frequency of bilateral involvement and posterior circulation involvement, and the homozygous p.R4810K mutation was associated with early-onset of the disease (17–19). The p.A4399T polymorphism, which is common in Chinese patients, is associated with hemorrhagic MMD (odds ratio = 2.8; 95% confidence interval, 1.2–6.5) (20). An intronic variant, rs9916351, was reported to be associated with early onset MMD in a Chinese population (21).

The p.R4810K mutation has been reported to be associated with various vascular phenotypes. First, it was shown to be associated with quasi-MMD (syndromic MMD) (22), moyamoya angiopathy with a known comorbidity such as Down syndrome or neurofibromatosis type I (NF1) (23). In addition, it was associated with other vascular diseases including non-moyamoya cerebral arteriopathy (24–26), coronary artery disease (7, 8) and pulmonary artery hypertension (9, 27). The p.R4810K mutation was also associated with hypertension in a dominant fashion (28), while rs9916351 was associated with hypertension in a recessive fashion (29).

Loci on 4q.32.1, 10q23.31, and Xp28 have been identified as loci for quasi-MMD, and *GUCY1A3, ACTA2*, and *BRCC3* was found to be a disease associated gene for each locus (11, 30, 31). Genetic analysis of three families with autosomal recessive mode of inheritance identified *GUCY1A3* as a cause of moyamoya angiopathy with achalasia, esophageal sphincter muscle contraction (32). Interestingly, some patients with moyamoya angiopathy associated with *GUCY1A3* mutation do not have achalasia (12), meaning that the patient can be diagnosed as MMD but not quasi-MMD. In *RNF213*, the p.R4810K mutation was common to various vascular phenotypes including MMD, coronary artery disease and pulmonary artery hypertension, whereas different mutations were found in each phenotype for *GUCY1A3*. In detail, nonsense mutation p.R349X, p.E391KfsX, and c.1086 +1G>A splice donor site mutation were associated with moyamoya with achalasia (11). *GUCY1A3* rs1842896 polymorphism was a risk factor for large artery arteriopathy stroke in a Southern Han Chinese population (33). Loss of function mutation p.L163FfsX was associated with an increased risk of myocardial infarction (13). Gain of function mutation p.A681T was associated with a reduced risk of pulmonary artery hypertension (14). A SNP, rs13139571, at the *GUCY1A3*-*GUCY1B3* locus was associated with high blood pressure (34). Thus, different mutations or SNPs exhibit different phenotypes, although hypertension is a common phenotype for most mutations and polymorphisms in *GUCY1A3*.

## Molecular Structure and Functions

### Molecular Structure and Functions of RNF213/Mysterin

**Figure 1:**
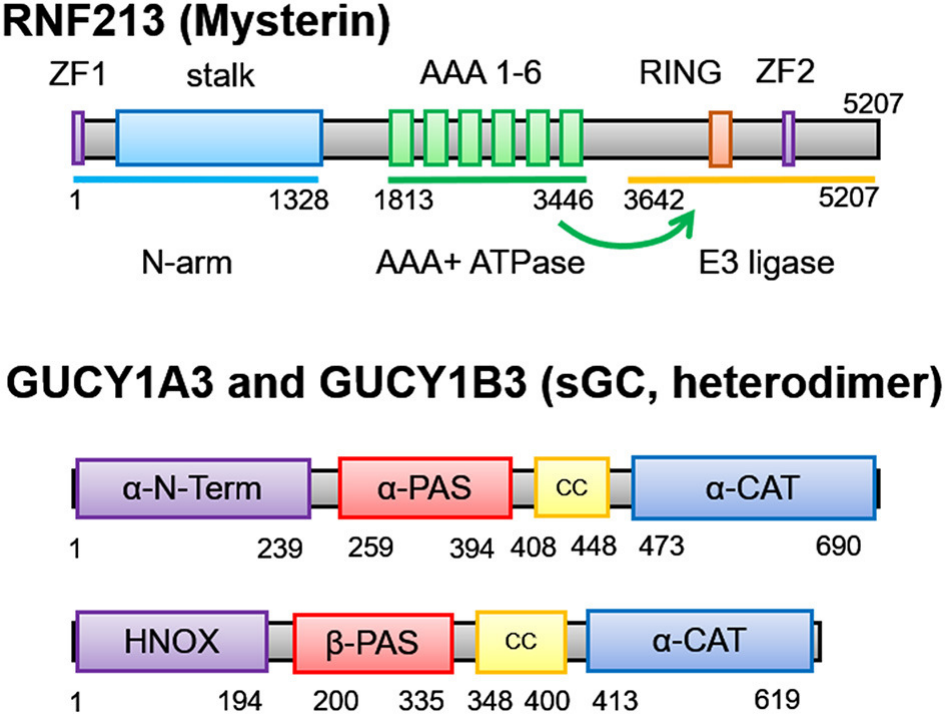
Molecular structure of RNF213 and soluble guanylate cyclase (sGC) encoded by GUCY1A3. RNF213 consists of N-arm, AAA+ ATPase, and E3 ligase. E3 ligase contains two different types of zinc finger, RING and ZF2 (NFX1-type zinc finger). Not only mutations in the RING and ZF2, but also mutations in the AAA+ ATPase may disturb the function of E3 ligase. GUCY1A3 and GUCY1B3 encode the α1 and β1 subunits of soluble guanylate cyclase (sGC) to form a heterodimer.

RNF213/Mysterin is a unique protein which has both functional AAA+ ATPase and E3 ligase. It is a large protein consisting of 5,207 amino acids with a size of 591-kDa (Figure 1). Isoform 3 which lacks exon 4 is the major isoform (NP_001243000.2) (4), and the p.R4810K mutation (rs112735431) is assigned as c.14429G>A. A recent cryo-EM analysis revealed the molecular structure of RNF213 (35, 36). It consists of three structural components including N-terminal structural motif (N-arm), AAA+ ATPase with Walker motif A and B, and E3 ligase with RING finger and NFX1-type zinc finger (Figure 1). Mutations associated with MMD are dominantly observed in E3 ligase.

**Figure 2:**
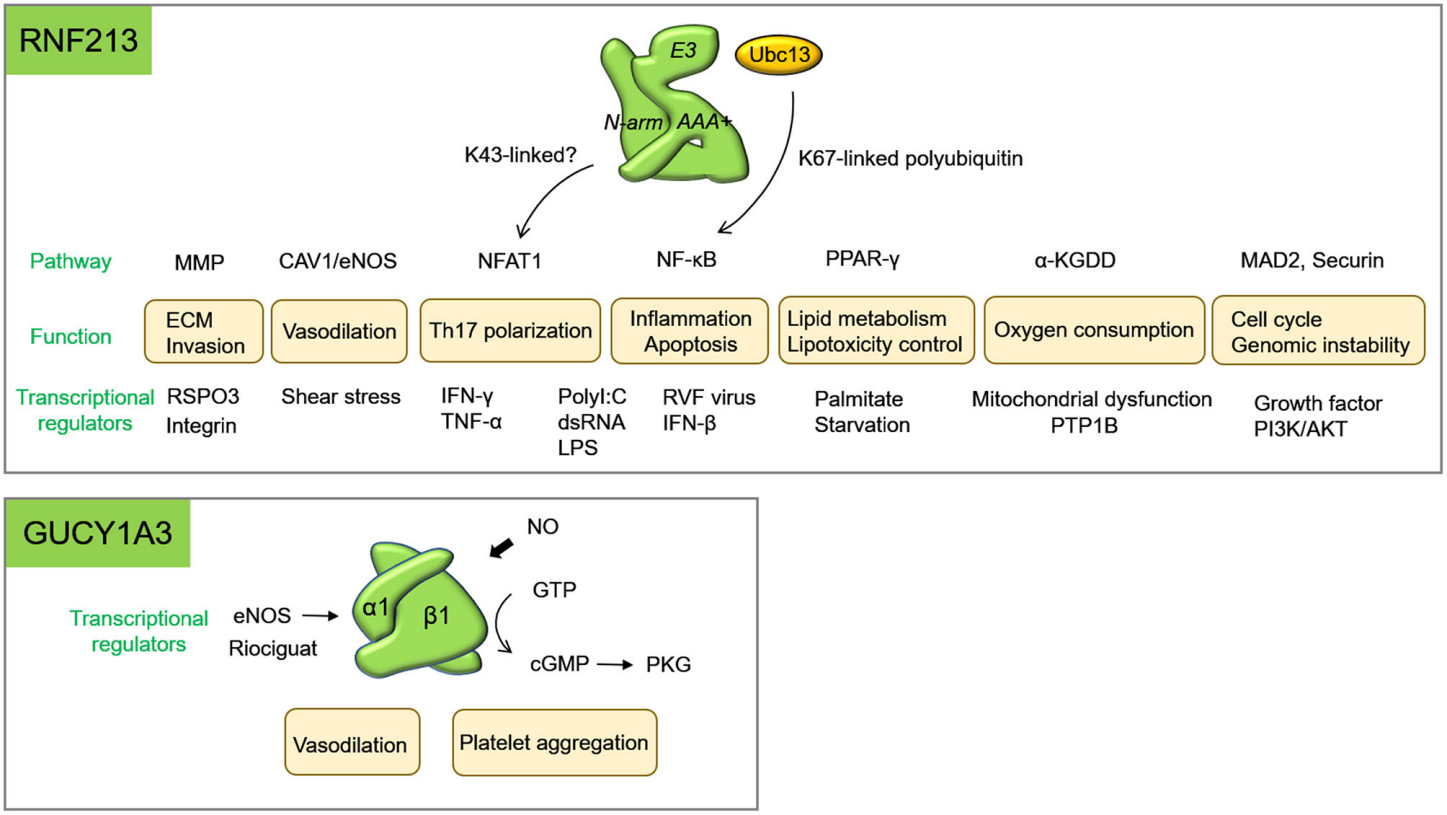
Diverse biological functions of RNF213 and GUCY1A3. Biological functions and their related molecules were shown. Transcriptional regulators of RNF213 for each function were listed below. α-KGDDs represents α-Ketoglutarate-dependent dioxygenases; ECM, extracellular matrix; PDE, phosphodiesterase; RVF, Rift Valley fever.

E3 ligase plays a central role in post-translational modification by ubiquitination. It recruits an E2 ubiquitin-conjugating enzyme that has been loaded with ubiquitin, recognizes a protein substrate and assists or directly catalyzes the transfer of ubiquitin from the E2 to the protein substrate. Ubiquitin contains seven lysine (K) residues that, together with its amino terminus, provide eight attachment sites for further ubiquitin molecules (K0, K6, K11, K27, K29, K33 or K48, K63), thereby allowing the formation of polymeric chains (37). Variety of combination of branch sites and the length of polyubiquitin chain allow the system to be highly complex, and the system is called “ubiquitin codes.” Together with its various substrates, ubiquitin acts as a versatile cellular signal that controls a wide range of biological processes including protein degradation, DNA repair, endocytosis, autophagy, transcription, immunity, and inflammation. K63 linkages are known to regulate activation of the nuclear factor-kappa B (NF-κB) transcription factor, DNA repair, innate immune responses, clearance of damaged mitochondria, and protein sorting (38). RNF213 RING domain cooperates with Ubc13 E2 ubiquitin-conjugating enzyme to generate K63-linked polyubiquitin chains and induces NF-κB activation (Figure 2). MMD-associated mutations in the RING domain enhances NF-κB activation (39). Still, ubiquitination targets of RNF213 remain largely unknown, except for Nuclear factor of activated T-cells 1 (NFAT1) and filamin A (40).

The AAA+ superfamily of ATPases has diverse cellular functions including membrane fusion, proteolysis and DNA replication, and it works as molecular chaperon (41). RNF213 was considered to belong to the dynein ATPase family which has 6 AAA domains (35). It forms hexamer (42) and is similar to the resting states of dynein. However, it does not have motor activity along the microtubule but has trans-thiolation activity, which transfers ubiquitin from one substrate to another (36). This means that ATPase and E3 ligase work cooperatively. In fact, deletion or mutations of the ATPase domain reduced the function of E3 ligase (39, 43) (Figure 1). This may explain the reason why MMD is caused by both mutations in the ATPase and those in the E3 ligase.

### Molecular Structure and Functions of Soluble Guanylate Cyclase Encoded by *GUCY1A3*

**Figure 3:**
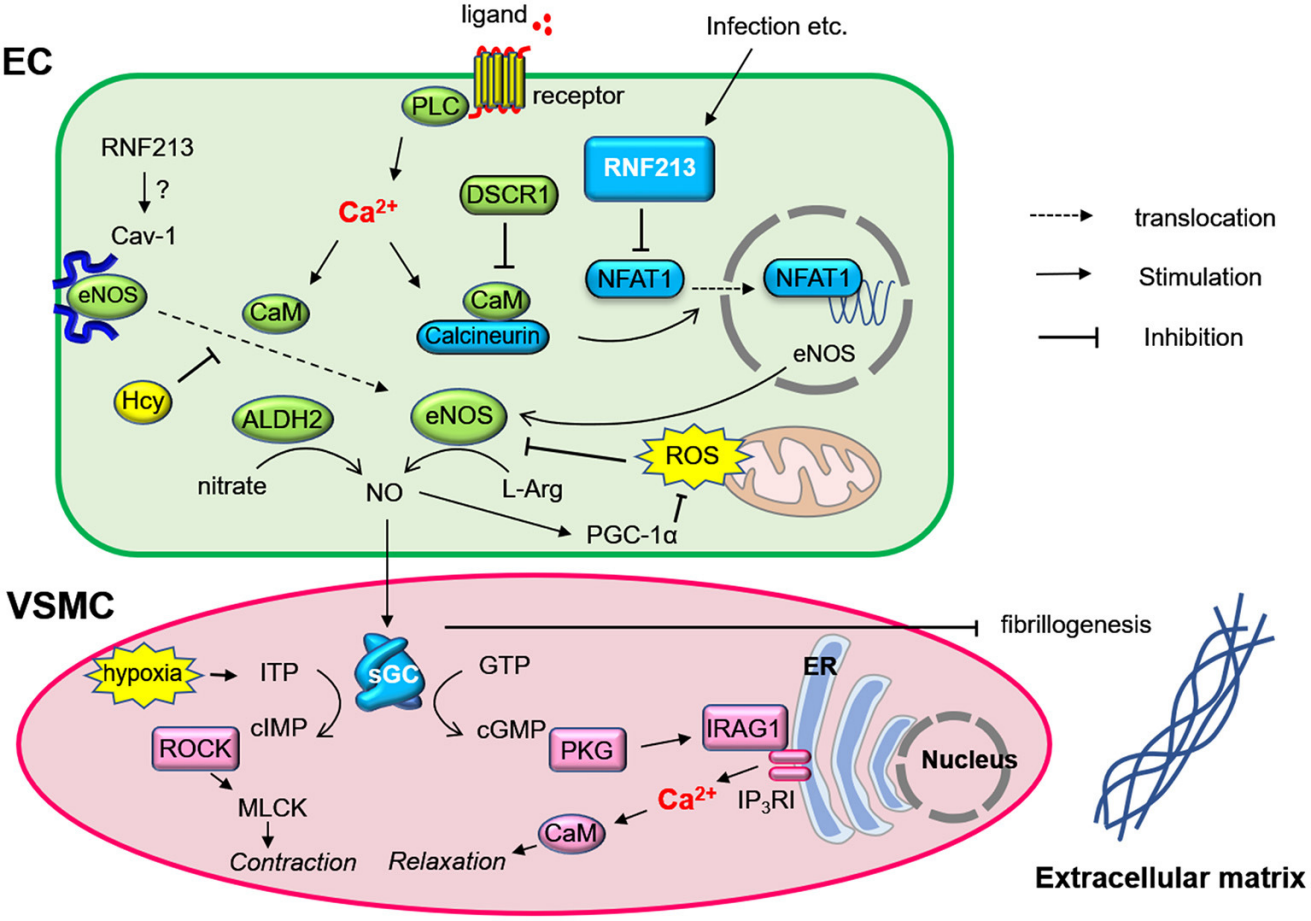
Regulation of nitric oxide signaling by RNF213 (hypothetical role) and GUCY1A3 (sGC) in endothelial cells and vascular smooth muscle cells. Endothelial cell stimulation induces calcium-calmodulin-calcineurin signaling, which produces eNOS via NFAT1. RNF213 degrades NFAT1, which may regulate eNOS production. NO is synthesized from L-Arginin by eNOS and diffuses to vascular smooth muscle cells. NO is catalyzed by soluble guanylate cyclase (sGC), and cIMP or cGMP transmit signaling to induce vasodilatation. sGC also contributes to prohibiting fibrinogenesis. Many molecules known to be associated with moyamoya disease such as caveolin-1 (CAV1), Down Syndrome Critical Region 1 (DSCR1), inositol 1,4,5-Triphosphate Receptor Associated 1 (IRAG1) is involved in this molecular network. ALDH2, aldehyde dehydrogenase 2; CaM, calmodulin; cIMP, Inosine 3', 5'-cyclic monophosphosphate; EC, endothelial cell; Hcy, homocysteine; ITP, inosine triphosphate; L-Arg, L-Arginin; MLCK, myosin light-chain kinase; PKD, protein kinase G; PGC-1α, PPARγ-coactivator-1α; PLC, phospholipase C; ROCK, Rho kinase; ROS, reactive oxygen species; VSMC, vascular smooth muscle cell.

*GUCY1A3* encodes the α1 subunit of soluble guanylate cyclase (sGC), which forms heterodimeric enzyme with ß1 subunit encoded by *GUCY1B3* (Figure 1), and it is the major receptor for nitric oxide (NO) (Figures 2, 3). NO binds the heme iron of sGC to induce production of cyclic guanosine monophosphate (cGMP), which then activates the cGMP-dependent protein kinase (PKG) pathway (44). *Murine retrovirus integration site 1* (*MRVI1*) encodes Inositol 1,4,5-Triphosphate Receptor Associated 1 (IRAG1) and plays a role as NO/PRKG1-dependent regulator of IP3-induced calcium release. Phosphorylation of *MRVI1* by PRKG1 inhibits bradykinin and IP3-induced calcium release from intracellular stores, leading to inhibition of platelet activation and aggregation. It also mediates NO-dependent inhibition of calcium signaling, which contributes to NO-dependent relaxation of smooth muscle cells. Intrudingly, *MRVI1* mutation was found to increase the risk of developing moyamoya angiopathy in patients with NF1. Specifically, p.P186S substitution (rs35857561) in *MRVI1* was segregated with quasi-MMD in both the Italian and German NF1 families (45).

The p.C517Y mutation in *GUCY1A3* was found in patients with MMD without any evidence of achalasia (12). Sf9 cells expressing the *GUCY1A3* p.C517Y mutation showed lower basal cGMP-forming activity and lower maximal NO-induced activity. Thus, the p.C517Y missense mutation led to a significantly blunted response in NO signaling and decreased cGMP production. This was in consistent with previously published observations on rat sGC-containing nitrosylated C516 (mouse homolog to human C517) to resulted in desensitization of sGC to NO activation (46).

## Transcriptional Regulation

### Transcriptional Regulation of *RNF213*: A Sensor for Mitochondrial Damage and Inflammation

Various factors have been reported to upregulate *RNF213*, and all of them were related to inflammation caused by mitochondrial dysfunction and infection. In patients with MMD, morphological abnormalities of mitochondria with higher reactive oxygen species (ROS) as well as elevated Ca2+ levels and reduced mitochondrial reductase activity was detected in circulating endothelial colony-forming cells (47). Recent evidence suggests that mitochondrial dysfunction upregulates *RNF213* (Figure 2). Genetic ablation of several mitochondrial matrix factors such as the peptidase ClpP, the transcription factor Tfam increased *Rnf213* expression in various organs in mice (48). *RNF213* is also upregulated by poly(I:C), which triggers toll like receptor 3 (TLR3)-mediated responses to double-stranded RNA (dsRNA) toxicity (48) (Figure 2). Since dysfunctional mitochondria were recently reported to release immune-stimulatory dsRNA into the cytosol, RNA-dependent inflammation initiated by mitochondrial dysfunction or infections may increase the penetrance of patient mutations in *RNF213*. *SAMHD1* loss of function mutations is known to cause inflammatory vasculopathy including moyamoya angiopathy. It is noteworthy that SAMHD1 works as a sensor of dsRNA and dsDNA at replication fork (49), and dysfunction of this gene is associated with excess interferon (IFN) production and senescence associated secretory phenotype (SASP). Tocilizumab, an interleukin (IL)-6 antagonist, was effective to reverse cerebral vasculopathy in a patient with homozygous *SAMHD1* mutation (50). Therefore, regulation of inflammatory signals may be effective for *RNF213*-related disease as well.

In endothelial cells, *RNF213* is also upregulated by type I IFN (51), and the combination of IFN-γ and tumor necrosis factor (TNF)-α synergistically activated transcription of *RNF213* both *in vitro* and *in vivo* (52) (Figure 2). The transcription of *RNF213* was regulated by phosphatidylinositol-3 kinase (PI3K)/AKT and dsRNA-dependent protein kinase R (PKR) pathways. Key et al. also showed that PKR inhibitor inhibited *RNF213* expression; however, the effect was only seen in neuronal cell lines but not in fibroblast or HUVEC (48). These findings suggest that transcriptional regulation of *RNF213* seems to be cell type- and context-dependent. In macrophage or adipocyte, another inflammatory component, lipopolysaccharide (LPS), was shown to upregulate *RNF213*, while peroxisome proliferator-activated receptor γ (PPARγ), an anti-inflammatory signal, downregulated it (53). In adipocyte, PTP1B mediated higher expression of *RNF213* by TNF-α, while PTP1B suppresses E3 ligase activity of RNF213 in a breast cancer cell line under hypoxic condition (54) (Supplementary Figure 1). Again, regulation of *RNF213* was cell-type and context-dependent.

### Transcriptional Regulation of *GUCY1A3*

As compared with *RNF213*, transcriptional regulation of *GUCY1A3* is not well-characterized. Investigation of the promoter activity of *GUCY1A3* identified several consensus sequences including NFAT and NF-κB (p50) (55). Because both NFAT and NFκB are master regulators of inflammation, *GUCY1A3* may also be regulated by inflammatory signals as is the case with *RNF213*. Another regulator of *GUCY1A3* is eNOS. It has been reported that *GUCY1A3* is upregulated by eNOS in the pulmonary vasculature (56). Upon chemical stimulation of sGC, Zinc finger E box-binding homeobox 1 transcription factor (ZEB1) binds to the promoter region of GUCY1A3 (57).

## Functions of RNF213 and sGC in the Vascular Wall and Circulation

### Histopathological Features of MMD

Histopathological features of MMD were intimal fibrous thickening without significant inflammatory cell infiltration or lipids, the tortuous internal elastic lamina, and disrupted ECs (58, 59). VSMC proliferation was seen in the internal carotid artery, whereas it was atrophic in the distal part of the lenticulostriate arteries (59). Moth-eaten change of the cells with increased extracellular matrix (ECM) was observed in the media, suggesting irregular atrophy and damage of the smooth muscle cells. We will discuss a possible link between these histopathological characteristics and the molecular functions of RNF213 and sGC.

### Functions of RNF213 in ECs and VSMCs

Effect of the p.R4180K mutation in endothelial cells were first reported by Hitomi et al. who compared iPS-derived ECs from patients with MMD and unaffected carrier with the p.R4810K mutation and control individuals with wild type *RNF213*. Angiogenic ability (tube formation) of iPS-ECs from patients and mutation carriers were lower than those from wild-type subjects (60). Gene expression profiles showed that Securin was down-regulated, and knock-down of Securin also impaired the angiogenic activity. *RNF213* p.D4013N, p.R4019C, and p.V4146A variants transfected into human umbilical vein endothelial cells had significantly decreased migratory abilities (61).

Effect of the p.R4810K in VSMCs were reported by Tokairin et al. By using VSMCs from iPS-derived neural crest stem cells, they showed that there was no significant difference in VSMC markers, cellular proliferation, migration, or contractile abilities between patients with the p.R4810K mutation and controls (62). Gene expression patterns of iPS-derived VSMCs were similar between patients and controls, whereas iPS-derived ECs displayed distinct patterns. Therefore, they speculated that pathological traits can be driven by naïve ECs predominantly and that VSMCs may require specific environmental factors. In agreement with this concept, RNF213 deficiency affected the cell growth of ECs but not VSMC or fibroblast (52). Likewise, peripheral blood-derived endothelial colony-forming cells but not smooth muscle progenitor cells were responsible for disturbed angiogenic activity when these cells from patients with MMD and healthy controls were co-cultured (63). Taken together, ECs is more likely to contributed to the pathogenesis of MMD, but this model cannot explain the damaged VSMCs as observed in patients with MMD. Therefore, there remains the possibility that even minimum changes in VSMCs caused by RNF213 mutation may affect the phenotype in long-term observation because MMD is a chronically progressive disease.

Although fibrous thickening is a distinguished pathological feature of affected arteries of patients with MMD, little is known about fibrosis in MMD. Hamauchi et al. showed that extracellular matrix (ECM) receptor-related genes, including integrin β3, were significantly downregulated in iPS-derived ECs from patients with the p.R4810K mutation (64). Masuo et al. investigated extracellular matrix secreted from ECs. They showed that iPS-derived ECs from patients with MMD produces less chondroitin sulfate as compared with those from controls (65). Because different types of chondroitin sulfate act differently on fibrillogenesis (e.g., versican enhances fibrillogenesis, while aggrecan, decorin, and lumican have the opposite effect), it remains unclear whether the reduced amount of chondroitin sulfate is associated with increased fibrosis in patients with MMD. Perivascular adipose tissue (66–68) is a well-known source of fibrous tissue in vascular disease, but it is scarce in the intracranial arteries. Thus, VSMCs and their dedifferentiated form are postulated to be responsible for fibrosis, and ECs may have some role in regulating fibrillogenesis of VSMCs via secretion of MMPs and ECM.

### Functions of sGC in ECs, VSMCs, and Platelets

In contrast to the major role of RNF213 in ECs, sGC plays an important role in VSMC relaxation and inhibition of platelet aggregation (69). NO-driven cGMP production exerts an anti-atherogenic effects, including vasodilatation, inhibition of vascular smooth muscle proliferation, blockade of leukocyte recruitment, and anti-platelet activity. Although functions of *GUCY1A3* mutation were not tested in VSMCs from patients with MMD, influence of sGC dysfunction in aortic or pulmonary smooth muscle cells have been well-studied. A single nucleotide polymorphism in *GUCY1A3*, rs7692387, was associated with coronary artery disease at genome-wide significance, and it interferes with binding of the transcription factor ZEB1 and impairs *GUCY1A3* expression, leading to lower sGC levels and lower sGC activity after stimulation (57). Accordingly, inhibitory effects of sGC stimulator on VSMC migration were abolished in homozygous risk allele carriers. In agreement with these human data, lower *Gucy1a3* expression correlated with more aortic atherosclerosis in a population of genetically diverse mice (70). Interestingly, sGC stimulation also has an anti-fibrotic effect (71). Therefore, dysfunctional mutations in *GUCY1A3* may be a cause of fibrous thickening of intima in moyamoya angiopathy. *In vitro* and *in vivo* models of moyamoya angiopathy having the *GUCY1A3* mutation should be developed and investigated.

NO and sGC are generally recognized as major regulators of platelet functions (72) (Figure 2). NO mediates inhibition of collagen-induced platelet aggregation and secretion via sGC. Hervé et al. compared platelet function between patients with moyamoya angiopathy with *GUCY1A3* mutation and control individuals. Bleeding time and platelet count was not different, but inhibition of collagen-induced platelet aggregation and secretion by NO donor (PROLI NONOate or sodium nitroprusside) was significantly impaired in platelets derived from patients (11). The risk allele of coronary artery disease, rs7692387 in *GUCY1A3*, also impaired an inhibitory effect of platelet aggregation by NO donor. In agreement with human data, loss of the α1 subunit of sGC in mice leads to enhanced thrombus formation (13).

Functions of sGC are mostly studied in VSMCs and platelets, but sGC also plays a role in ECs. Pyriochou et al. demonstrated that overexpression of sGC promotes EC proliferation, migration, and tube-like network formation (73). Interestingly, pharmacological stimulation of PKG, a downstream signaling molecule of sGC-cGMP, actually have effects on ECs, VSMCs and platelets. It reduces neointimal hyperplasia, inhibits platelet aggregation, and facilitates re- endothelialization (74).

## Functions of RNF213 and sGC in Vascular Insults

### Vascular Insults Associated With MMD

As discussed above in the section of transcriptional regulation, *RNF213* is upregulated by mitochondrial damage, LPS, poly I:C or type I IFNs, and *GUCY1A3* is regulated by NF-κB and NFAT. This suggests that vascular insults such as infection or hypoxia should be associated with moyamoya angiopathy. In fact, recent evidences suggest that dyslipidemia, an elevated level of homocysteine and viral or bacterial infection have an impact on the progression of MMD. Therefore, it is important to clarify the functions of RNF213 and sGC in association with these vascular insults.

### Dyslipidemia and Lipotoxicity

Association of lipid metabolism and MMD has been reported very recently. Ge et al. reported that the HDL cholesterol level was inversely associated with the risk of MMD (75). Church et al. reported that dyslipidemia was a risk factor for contralateral progression in patients with unilateral MMD (76). Hirano et al. also reported that dyslipidemia was associated with symptomization of asymptomatic patients with MMD (77). These findings suggest that lipid metabolism may be involved in the pathogenesis of MMD, although neither study showed direct association between RNF213 mutations and dyslipidemia.

In this context, functions of RNF213 in lipid metabolism are of particular interest (Supplementary Figure 1). Strikingly, it has been reported that RNF213 accelerate triglyceride accumulation in lipid droplet (LD) by eliminating adipose triglyceride lipase (ATGL) from LD, one of the major lipolytic molecule of triglyceride (42). The authors showed that the ubiquitin ligase and ATPase activities of RNF213 were both important for its proper LD targeting and its fat-stabilizing activity. Specifically, localization of RNF213 on lipid droplet and its fat-stabilizing activity were disturbed when Caucasian cysteine/histidine mutations (e.g., p.C3997Y) in the RING finger domain or mutations in the AAA+ ATPase domain were introduced. Of note, the p.D4013N mutation in the RING domain and p.R4810K in the E3 core at the C-terminal region, which are mutations found in familial MMD, did not alter the functions of RNF213. This may partly explain the mechanism of low penetrance of the p.D4013N and p.R4810K mutations and high penetrance of the cysteine/histidine mutations.

Lipotoxicity represents toxic effects by saturated fatty acid such as palmitate or stearate, and lipotoxic effect of palmitate on β cells in pancreas can be a cause of diabetes mellitus. It has been reported that ablation of RNF213 decreased lipotoxicity by palmitate (78). Lipid droplet itself does not exert lipotoxicity (79), while it reduces lipotoxicity through incorporating saturated fatty acid inside (80). In fact, RNF213 deficiency increases the activity of SCD1, a key enzyme that promotes palmitate detoxification and storage into triglycerides (78) (Supplementary Figure 1). Consistent with the protective effect against lipotoxicity, ablation of *Rnf213* recovered insulin levels in Akita mouse, a diabetes model with impaired insulin production, and it improved glucose tolerance by protecting islet β cells (81). In patients with MMD, the p.R4810K mutation was inversely associated with diabetes mellitus (odds ratio = 0.35; 95% confidence interval, 0.18–0.68) (82). Inverse association of p.R4810K with diabetes was also found in patients with coronary artery disease (odds ratio = 0.34; 95% confidence interval, 0.12–0.79) (7). In respect of protection from diabetes, the p.R4810K is considered to have loss-of-function or dominant negative properties.

### Vascular Insults by Homocysteine

Hypehomocysteinuria has been recognized as a cause of quasi-MMD, and recent study showed that homocysteine level is associated with an increased risk of MMD, especially for unilateral MMD (75). Duan et al. showed that rs9651118 in *Methylenetetrahydrofolate reductase* (*MTHFR*) gene and rs9651118 in *Transcobalamin 2* (*TCN2*) gene was associated with a homocysteine level, and they were also associated with a risk of MMD (21). Alcohol intake or folate deficiency is associated with increased levels of homocysteine (Supplementary Figure 1), and it has recently been reported that daily alcohol drinking was an independent risk factor for contralateral progression of unilateral MMD and daily drinker with the p.R4810K mutation had significantly higher risk of progression (83). Homocysteine decreased the expression of *CAV1* in coronary artery ECs, which induced translocation of NO from caveolae to non-caveolae fractions (Figure 3). As a result, homocysteine impairs NO release from ECs (84), leading to vascular dysfunction. Homocysteine has been shown to induce ER stress and apoptosis in a variety of cell types. Jeong et al. demonstrate that pharmacological inhibition of sGC almost completely abolished the protective effects of NO and nitrite, whereas pharmacological elevation of cellular cGMP, mimicked the protective action of NO donors in neurons (85). Thus, NO donors inhibit homocysteine-induced ER stress and apoptosis via NO-sGC-cGMP signaling pathway. In relation to the signaling of RNF213, Hcy upregulates *PTP1B* (86).

### Viral and Bacterial Infection

Infection has been postulated as a risk factor for MMD, although there is no solid evidence to prove it. Quasi-MMD associated with meningitis was reviewed by Mikami et al. It was estimated to be 2.2% of all the quasi-MMD (87), and it was caused by various pathogens. Among them, varicella zoster virus infection draws attention due to the high frequency of reversible arteriopathy called transient cerebral arteriopathy (TCA) or focal cerebral arteriopathy (FCA), which sometimes progresses to moyamoya (88). Pathological examination of postinfectious vasculopathy with progression to moyamoya angiopathy following Streptococcus pneumoniae meningitis was reported by Czartoski et al., and they showed that inflammation or atherogenic features were absent in the lesion. Due to the progressive course, elevated anti-β2-glicoprotein 1 IgG titers, and transient response to immunomodulatory therapy, they speculated that the vasculopathy was likely to be mediated by an autoimmune process (89).

Involvement of RNF213 in viral infection has just recently been reported. Homozygous deletion of *Rnf213* showed significantly shorter survival in C57BL/6 J mice lethally infected with Rift Valley fever virus (90), an enveloped negative single stranded RNA viruses. The mechanism of entry is dynamin-dependent, CAV1-mediated endocytosis (91). Because Rift Valley fever virus infection suppresses the response of IFN-β or other IFN-related molecules (92, 93), type-I IFN mediated upregulation of *Rnf213* is not likely to be the mechanism of action against the virus. More recently, it has been shown that RNF213 restricts the proliferation of cytosolic Salmonella and is essential for the generation of the bacterial ubiquitin coat, which initiates antibacterial autophagy (43). The ubiquitylation of LPS on Salmonella requires the AAA+ ATPase domain and newly identified NFX1-type zinc finger domain (ZF2 in Figure 1). Together with the fact that *RNF213* is upregulated by LPS, RNF213 would contribute to antibacterial immune reactions. However, no patient mutation tested in the study including p.R4810K mutation affected the ubiquitination of LPS.

## Other Properties

### Inflammation *via* NF-κB Signaling

RNF213 protects cells from ER stress and inflammation by lipotoxicity. Depletion of RNF213 stabilizes ER stress gene expression, normalizes the cellular lipidome, and blocks NF-κB pathway during palmitate exposure (78). Recent studies showed that RNF213 selectively cooperates with Ubc13 (E2 enzyme) to generate K63-linked polyubiquitin chains, but not K48-linked ones (39, 94). K63 linkages are known to regulate activation of the NF-κB transcription factor, DNA repair, innate immune responses, clearance of damaged mitochondria, and protein sorting (38). Interestingly, BRCC3, whose deletion was associated with X-linked syndromic moyamoya, is a E3 ligase that specifically cleaves K63-linked polyubiquitin chains (31). This molecule regulates the abundance of these polyubiquitin chains in chromatin and plays a role in the DNA damage response.

Importantly, most mutations in the RING domain found in patients with MMD reduced E3 ligase activity and many of them induced NF-κB activation (39). These mutations that induce NF-κB activation included not only Caucasian cysteine/histidine mutations but also proline mutations (p.P4007R in a Chinese patient and P4033L in a Caucasian patient). These mutations also induced apoptosis in a NF-κB dependent manner. However, p.D4013N mutation did not affect either E3 ligase activity or NF-κB activation. Importantly, critical point mutations in both the Walker A and B of the AAA domains, completely abrogated NF-κB activation by *RNF213* mutation in the RING domain. Thus, inflammation via NF-κB pathway was enhanced by patient mutations in *RNF213*, while it was suppressed in the absence of *RNF213*. In respect of NF-κB activation, *RNF213* mutations should have gain of function properties.

RNF213 also controls mitochondrial functions, cell cycle, or differentiation and maturation of immune cells (Figure 2, details are described in Supplementary Material). These properties will also play a role in the development of MMD.

## Potential Mechanism in MMD

### Possible Interaction of RNF213 and sGC

**Figure 4:**
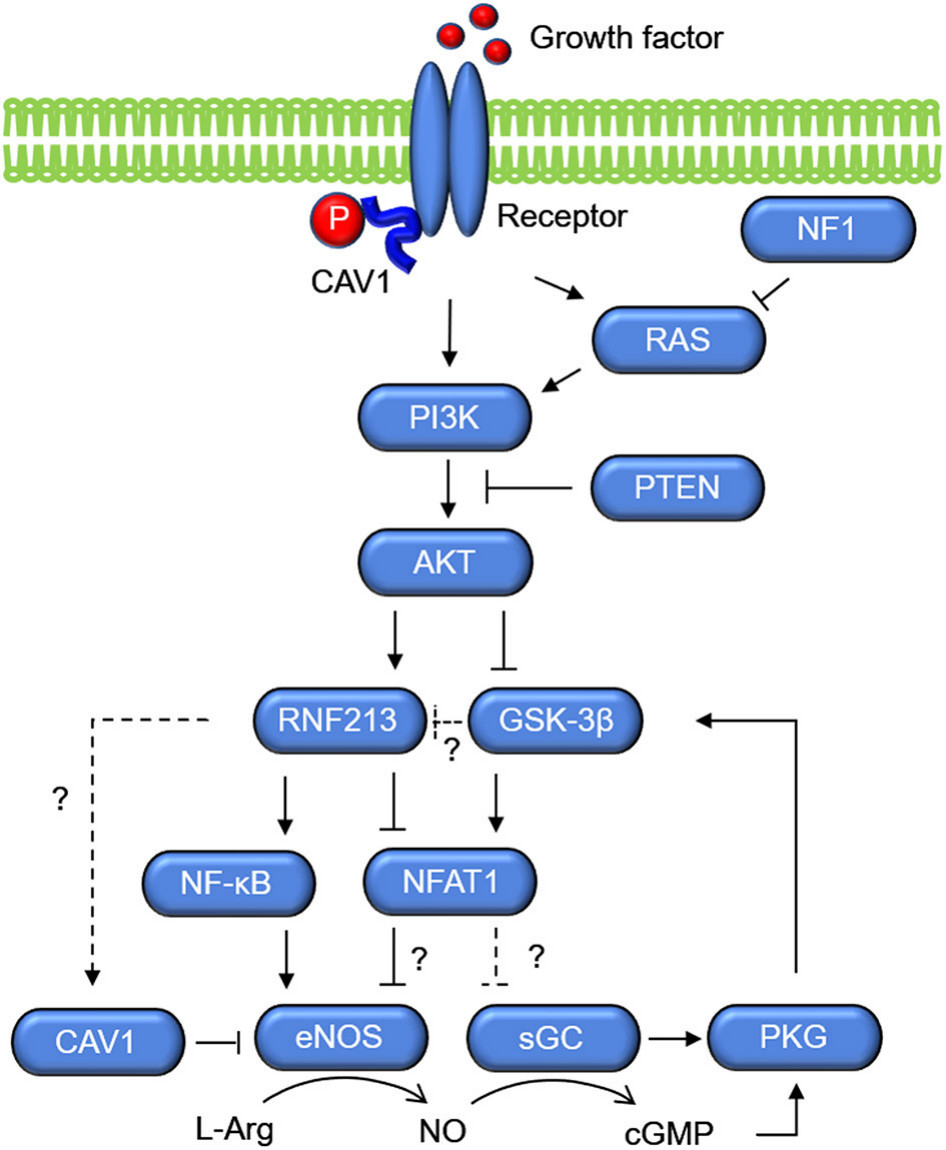
Molecular networks of RNF213 and GUCY1A3 (sGC). Growth factors activate PI3K/AKT, which upregulate the expression of RNF213 and eNOS. Activation of PI3K/AKT leads to inactivation of GSK-3β, which increases degradation of NFAT1 by the proteasome possibly via ubiquitination by RNF213. As opposed to the Ca+/Calmodulin/NFAT1 signaling shown in Figure 3, NFAT1 is assumed to suppress eNOS expression in the context of PI3K/AKT. GSK-3β, glycogen synthase kinase-3β; PKG, protein kinase G; sGC, soluble guanylate cyclase.

Molecular networks that potentially connects RNF213 and sGC are shown in Figure 4. The key molecule is NFAT1, which is a ubiquitin target of RNF213 downstream of non-canonical WNT/Ca^2+^ signaling (40). Activation of calcineurin/NFAT signaling by VEGF in human endothelial progenitor cells leads to increased eNOS protein expression and NO production (95). NFAT may also regulate the expression of sGC through binding of NFAT1 to the consensus sequence in GUCY1A3 (55). Activation of PI3K/AKT leads to inactivation of glycogen synthase kinase (GSK)-3β, which induces degradation of NFAT1 by the proteasome (96). Because PI3K/AKT was reported to be an upstream regulator of *RNF213* expression in ECs (52), the effect of PI3K/AKT on NFAT1 may be mediated by RNF213. Upregulation of *NFAT1* is also mediated by S-nitrosylation of RNF213 (97). S-nitrosylation is post-translational modification adding a nitrosyl group to the reactive thiol group of a cysteine to form S-nitrosothiol, which is a key mechanism in transferring NO-mediated signals. S-nitrosylation of ubiquitin ligase leads to its auto-ubiquitination and, as a consequence, increases its substrate levels. NFAT is, in turn, regulated by sGC (98) via activation of PKG, which phosphorylates GSK-3β.

Another key molecule that regulates NO signaling is caveolin. The integral membrane protein caveolin-1 (CAV1) is ~21–24 kDa and is found primarily in 50–100-nm flask-shaped invaginations called plasma membrane caveolae, where it acts as a scaffold to organize multiple molecular complexes that regulate a variety of cellular events. CAV1 is regulated by a signal mediated through Ca^2+^/calcineurin/NFAT (99). Importantly, CAV1 was reported to be associated not only with MMD, but also with coronary artery disease and pulmonary artery hypertension. The function of CAV1 is well-characterized in pulmonary artery hypertension (100–102). CAV1 level was decreased in patients with MMD as compared with either patients with intracranial atherosclerotic stroke or healthy subjects, and it was markedly decreased in *RNF213* R4810K variant carriers (103). However, it remains unclear whether RNF213 has direct, e.g., CAV1 is a target of ubiquitination by RNF213, or indirect association with CAV1. CAV1 is associated with eNOS release (Figure 4). NO is generated by eNOS release and it is metabolized by sGC. Direct binding of eNOS to the scaffolding domain of CAV1 is a well-accepted mechanism for inactivating eNOS (104). The absence of CAV1 is thought to promote eNOS dysfunction associated with cerebrovascular disease.

### Potential Mechanism of MMD

Based on the molecular functions and networks of RNF213 and sGC encoded by GUCY1A3 as discussed above, we propose a potential mechanism in MMD as shown in Figure 3. Under the physiological condition, EC stimulation by growth factors or shear stress induces Ca^2+^-calmodulin signaling. Calmodulin accelerates eNOS dissociation from CAV1 and upregulates eNOS production via Calcineurin/NAFT1. RNF213, which degrades NFAT1 through ubiquitin proteasome system, is not activated without pathological stimulations. eNOS then produces NO from L-Arginine, and NO diffuses into VSMCs. In VSMCs, NO activates sGC to produce cGMP. cGMP then activates PKG, which induces relaxation of VSMCs. Under the pathological condition where viral infection causes mitochondria destruction, *RNF213* is upregulated and prohibits eNOS production by degrading NFAT1. Patient mutations in *RNF213* may sustain the inflammation even after resolution of the infection, and it causes sustained cGMP signaling impairment. The same situation can be caused by *GUCY1A3* mutations. Impaired cGMP signaling leads to VSMC proliferation, impaired vasodilation, and fibrosis as well as endothelial dysfunction. These events will account for intimal hyperplasia with fibrous thickening, a typical pathological feature of MMD. Because impaired cGMP signaling can cause VSMC dedifferentiation and endothelial-to-mesenchymal transition, these cells can be potential sources of fibrosis.

Impaired protection from vascular insults is also a potential mechanism of arterial stenosis in MMD. As mentioned in the previous section, sGC acts protective against homocysteine, and RNF213 regulates lipotoxicity and has antiviral and antibacterial properties. Viral or bacterial infection causes type I IFN production and mitochondrial dysfunction, and they increase the *RNF213* expression. When patient mutations induce dysfunction of RNF213 or sGC, vascular damage caused by homocysteine, dyslipidemia or infection may be amplified, leading to chronic inflammation. *RNF213* mutations also induce inflammation via NF-κB. This may cause damaged VSMCs, which is another typical pathological feature of MMD.

In terms of inflammation, autoimmune conditions with Th17+ T cell polarization seem to be associated with MMD as discussed in the Supplementary Material. However, the effect of *RNF213* and *GUCY1A3* mutations have been studied mostly in vascular cells (ECs and VSMCs) but not in immune cells. To understand the precise mechanism of MMD, investigation of functions of RNF213 and GUCY1A3 in immune cells especially T cell, B cell, neutrophil and dendritic cell will be needed.

### Potential Therapeutic Strategy for MMD

Based on these potential mechanisms, we propose several therapeutic strategies that includes lipid and homocysteine regulation (Mediterranean or Japanese traditional diet, restriction of alcohol intake, and lipid/homocysteine lowering drugs), control of inflammation (avoidance of hypoxic condition and anti-inflammatory drugs), and pharmacological stimulation of eNOS-sGC-cGMP pathway. Candidates for pharmacological treatment of MMD would be anti-inflammatory drugs (e.g., COX-2 inhibitor or anti-IL-6 antibody), lipid lowering (e.g., statins or PCSK9 inhibitor) and homocysteine lowering drugs (e.g., folate or vitamin B12). Riociguat, a stimulator for sGC that is used to treat pulmonary artery hypertension, may be another option. However, there is not enough evidence that proves functionality and interaction of the candidate molecules, which account for the phenotypes of MMD. More precise molecular networks should be identified to develop a new treatment strategy.

## Conclusions

In 10 years after identification of *RNF213* as a susceptibility gene for MMD, function of RNF213 has been gradually unraveled. It plays a key role in lipid metabolism, oxygen consumption, cell cycle control and inflammation, and it contributes to the maintenance of vascular cells. GUCY1A3 is a regulator of platelet function and VSMC contraction via NO-sGC-cGMP pathway. Both mutations in *RNF213* and *GUCY1A3* cause not only MMD, but also non-moyamoya intracranial arterial diseases, coronary artery disease, and pulmonary artery hypertension. They have significant interaction with CAV1 and NFAT1, both of which have diverse molecular functions including immune regulation and cell cycle control. Functions of RNF213 and GUCY1A3 in ECs and VSMCs have been well-studied, but a precise mechanism of intimal thickening, fibrosis and its origin remain unresolved. Involvement of other vascular components such as inflammatory cells, platelet and extracellular matrix need to be further investigated.

## Author Contributions

YM conceived and designed the study, performed the literature search, and drafted the paper. SM reviewed and edited the manuscript. All authors contributed to the article and approved the submitted version.

## Conflict of Interest

The authors declare that the research was conducted in the absence of any commercial or financial relationships that could be construed as a potential conflict of interest.

## Publisher's Note

All claims expressed in this article are solely those of the authors and do not necessarily represent those of their affiliated organizations, or those of the publisher, the editors and the reviewers. Any product that may be evaluated in this article, or claim that may be made by its manufacturer, is not guaranteed or endorsed by the publisher.
