# Supplementary figures and images for: RNF213 and GUCY1A3 in Moyamoya Disease: Key Regulators of Metabolism, Inflammation, and Vascular Stability
Source: Front Neurol. 2021 Jul 26;12:687088. doi: 10.3389/fneur.2021.687088 (PMC8350054; doi:10.3389/fneur.2021.687088)

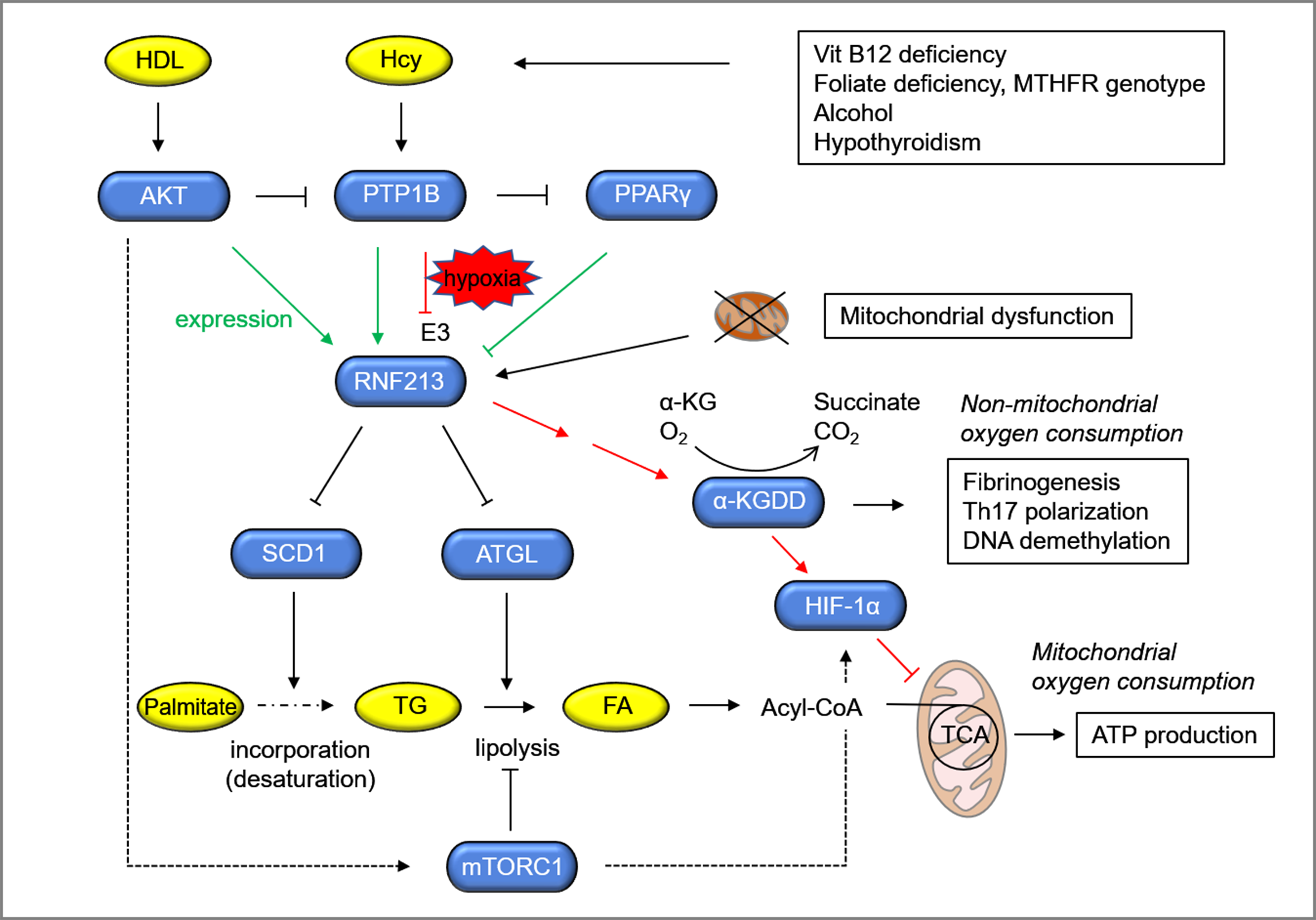

Supplement: Supplementary file 1 [file Image_1.TIF]
