# Supplementary material for: RNF213 and GUCY1A3 in Moyamoya Disease: Key Regulators of Metabolism, Inflammation, and Vascular Stability
Source: Front Neurol. 2021 Jul 26;12:687088. doi: 10.3389/fneur.2021.687088 (PMC8350054; doi:10.3389/fneur.2021.687088)
Supplement: Supplementary file 2 [file Data_Sheet_1.docx]

**Supplemental Materials**

# Additional properties of RNF213

## RFN213 regulates mitochondria damage and non-mitochondrial oxygen consumption

Proper regulation of cellular lipid storage and oxidation is indispensable for the maintenance of cellular energy homeostasis. Mitochondrial function has been shown to be a main determinant of functional lipid storage and oxidation (Veliova et al. 2020). It has been reported that RNF213 works as a regulator of non-mitochondrial oxygen consumption. *Protein tyrosine phosphatase 1B* (*PTP1B*) deficiency upregulate *RNF213*, and it sensitizes HER2+ breast cancer lines to hypoxia by increasing non-mitochondria oxygen consumption by α-Ketoglutarate-dependent dioxygenases (α-KGDDs). *RNF213* knockdown reverses the effects of *PTP1B* deficiency on α-KGDDs, non-mitochondrial oxygen consumption and hypoxia-induced death of HER2+ breast cancer cells (Banh et al. 2016). α-KGDDs use α-KG and oxygen as co-substrates to catalyse hydroxylation and demethylation reactions (Martínez-Reyes and Chandel 2020). Thus, in the presence of mitochondrial dysfunction, cancer cells in hypoxic condition increase the expression of *RNF213* and upregulates non-mitochondrial oxygen consumption. However, the functions of RNF213 under hypoxic condition in non-cancerous cells remains to be determined.

## RNF213 regulates cell cycle and its dysfunction induces genomic instability

RNF213 was also shown to play a role in cell cycle control (Figure 3). Overexpression of *RNF213* p.R4810K in HeLa cells inhibited cell proliferation and extended the time of mitosis 4-fold (Hitomi, Habu, and Kobayashi 2013). Immunoprecipitation revealed an RNF213/MAD2 complex, and the p.R4810K mutant formed a complex with MAD2 more readily than wild type. iPS-derived ECs from patients with MMD with the p.R4810K mutation and unaffected mutation carrier had significantly increased mitotic failure rates compared with controls. The resultant defects including mitotic abnormalities were considered to increase genomic instability and thus be risk factors for MMD. Another study showed that knock down of *RN213* decline in the proportion of cells in S-phase and increased that in G2/M phase in ECs (Ohkubo et al. 2015). In this context, p.R4810K seems to function as a loss-of-function mutation.

## RNF213 regulates differentiation and maturation of immune cells

*RNF213* is highly expressed in immune cells (Kamada et al. 2010) including T cells, B cells and neutrophils (Human Protein Atlas available from <http://www.proteinatlas.org>) (Uhlén et al. 2015). This is in line with the findings that patients with MMD elevated levels of autoantibody. Sigdel et al. performed high-density protein arrays to profile IgG autoantibodies in the sera from patients with MMD and they identified 165 significantly elevated antibodies (Sigdel et al. 2013). Among them, calmodulin kinases including CAMK2A, CAMK1D and CAMK2D, which has bidirectional interaction with NO-sGC-cGMP pathway, were listed among the top 30 ranked antibodies. Through a bioinformatics method, autoantibodies against APP, GPS1, STRA13, CTNNB1, ROR1 and EDIL3 were found to be significantly associated with MMD. It is noteworthy that CTNNB1 and ROR1 are the components of WNT signaling pathway, in which RSPO3 and RNF213 are involved.

In patients with MMD, the percentage of circulating Th17 cells as well as its dominantly secreting cytokines IL-17, TNF-α, IL-6 and IL-23 was significantly higher than those in patients with atherosclerosis or control subjects (Weng et al. 2017). Although the ratio of Th17 and regulatory T cells (Treg) was not different among the groups, the regulatory function of Treg was inhibited in patients with MMD. These observations are in agreement with the finding that patients with MMD show higher levels of autoantibodies, showing pro-inflammatory condition. In plasma proteome analysis, concentration of IL-17 receptor C (IL17RC) was significantly lower in MMD patients with the p.R4810K mutation as compared those without the mutation (Lee et al. 2019). In vivo analysis, the ratio of regulatory T cells, in response to strong immune adjuvant, was significantly lower in Rnf213-deficient mice than wild type (Kanoke et al. 2016). Double knockout of Tet1 and Tet2, members of α-KGDD that is positively regulated by RNF213, markedly increased the percentage of IL-17+Foxp3− cells (Th17 polarization), and decreased the percentage of IL-17−Foxp3+ cells (Xu et al. 2017). Recent report by Tashiro et al. showed that dendritic cells from Rnf213 knock-out mice or Rnf213-mutation knock-in mice (p.R4757K, the mouse homolog of human p.R4810K) had reduced capacity of antigen uptake or antigen-specific T cell activation (Tashiro et al. 2021). These lines of evidence suggest that RNF213 might regulate the balance between pro- and anti-inflammatory conditions (Figure 3). Further study is needed to elucidate the functions of RNF213 in immunity and inflammation in the vasculature.

**References**

Banh, Robert S., Caterina Iorio, Richard Marcotte, Yang Xu, Dan Cojocari, Anas Abdel Rahman, Judy Pawling, et al. 2016. “PTP1B Controls Non-Mitochondrial Oxygen Consumption by Regulating RNF213 to Promote Tumour Survival during Hypoxia.” *Nat Cell Biol* 18 (7): 803–13. https://doi.org/10.1038/ncb3376.

Hitomi, T, T Habu, and H Kobayashi. 2013. “The Moyamoya Disease Susceptibility Variant RNF213 R4810K (Rs112735431) Induces Genomic Instability by Mitotic Abnormality.” *Biochem Biophys Res Commun* 439 (4): 419–26. http://www.sciencedirect.com/science/article/pii/S0006291X13014149.

Kamada, Fumiaki, Yoko Aoki, Ayumi Narisawa, Yu Abe, Shoko Komatsuzaki, Atsuo Kikuchi, Junko Kanno, et al. 2010. “A Genome-Wide Association Study Identifies RNF213 as the First Moyamoya Disease Gene.” *J Hum Genet* 56 (1): 1–7. https://doi.org/10.1038/jhg.2010.132.

Kanoke, Atsushi, Miki Fujimura, Kuniyasu Niizuma, Taku Fujimura, Aya Kakizaki, Akira Ito, Hiroyuki Sakata, Mika Sato-Maeda, Shigeo Kure, and Teiji Tominaga. 2016. “Temporal Profile of Magnetic Resonance Angiography and Decreased Ratio of Regulatory T Cells after Immunological Adjuvant Administration to Mice Lacking RNF213, a Susceptibility Gene for Moyamoya Disease.” *Brain Research* 1642: 1–9. https://doi.org/10.1016/j.brainres.2016.03.009.

Lee, Ming-jen, Shannon Fallen, Yong Zhou, David Baxter, Kelsey Scherler, Meng-fai Kuo, and Kai Wang. 2019. “The Impact of Moyamoya Disease and RNF213 Mutations on the Spectrum of Plasma Protein and MicroRNA.” *J Clin Med* 8 (10): 1648.

Martínez-Reyes, Inmaculada, and Navdeep S. Chandel. 2020. “Mitochondrial TCA Cycle Metabolites Control Physiology and Disease.” *Nature Communications* 11 (1): 1–11. https://doi.org/10.1038/s41467-019-13668-3.

Ohkubo, Kazuhiro, Yasunari Sakai, Hirosuke Inoue, Satoshi Akamine, Yoshito Ishizaki, Yuki Matsushita, Masafumi Sanefuji, et al. 2015. “Moyamoya Disease Susceptibility Gene RNF213 Links Inflammatory and Angiogenic Signals in Endothelial Cells.” *Sci Rep* 5 (January): 13191. https://doi.org/10.1038/srep13191.

Sigdel, Tara K, Lorelei D Shoemaker, Rong Chen, Li Li, Atul J Butte, Minnie M Sarwal, and Gary K Steinberg. 2013. “Immune Response Profiling Identifies Autoantibodies Specific to Moyamoya Patients.” *Orphanet J Rare Dis* 8 (45): 1–11.

Tashiro, Ryosuke, Kuniyasu Niizuma, Jun Kasamatsu, Miki Fujimura, Atsuo Kikuchi, Yuko Okuyama, Sherif Rashad, Shigeo Kure, Naoto Ishii, and Teiji Tominaga. 2021. “Dysregulation of Rnf 213 Gene Contributes to T Cell Response via Antigen Uptake, Processing, and Presentation,” no. October 2020. https://doi.org/10.1002/jcp.30396.

Uhlén, Mathias, Linn Fagerberg, Bjö M. Hallström, Cecilia Lindskog, Per Oksvold, Adil Mardinoglu, Åsa Sivertsson, et al. 2015. “Tissue-Based Map of the Human Proteome.” *Science* 347 (6220). https://doi.org/10.1126/science.1260419.

Veliova, Michaela, Anton Petcherski, Marc Liesa, and Orian S Shirihai. 2020. “Seminars in Cell & Developmental Biology The Biology of Lipid Droplet-Bound Mitochondria.” *Semin Cell Dev Biol* 108 (December 2019): 1–10. https://doi.org/10.1016/j.semcdb.2020.04.013.

Weng, Leihua, Xiang Cao, Lijuan Han, Haoran Zhao, Shuwei Qiu, Yaping Yan, Xiaoying Wang, et al. 2017. “Association of Increased Treg and Th17 with Pathogenesis of Moyamoya Disease.” *Sci Rep* 7 (1): 1–8. https://doi.org/10.1038/s41598-017-03278-8.

Xu, Tao, Kelly M. Stewart, Xiaohu Wang, Kai Liu, Min Xie, Jae Kyu Ryu, Ke Li, et al. 2017. “Metabolic Control of TH17 and Induced Treg Cell Balance by an Epigenetic Mechanism.” *Nature* 548 (7666): 228–33. https://doi.org/10.1038/nature23475.

**Supplemental Figure**

**Supplemental figure 1. Metabolic functions of RNF213.** Mitochondrial dysfunction upregulates *RNF213*. Ablation of mitochondrial matrix factors increase the expression of RNF213. If regulation of RNF213 by PTP1B is abrogated, non-mitochondrial oxygen consumption via α-KGDD is accelerated, and tumor cells will die due to excessive oxygen consumption. RNF213 shifts the balance from mitochondrial oxygen consumption (ATP production by lipolysis of triglyceride) to non-mitochondrial oxygen consumption (NMOC) and triglyceride accumulation. Although direct effects of HDL and Homocystein (Hcy) on RNF213 remain unknown, AKT and PTP1B, which are affected by HDL and Hcy have regulatory role on RNF213. Suppression of RNF213 activates SCD1 and ATGL, which accelerate lipolysis and reduces lipotoxic effect by saturated fatty acids such as palmitate. FA, fatty acid; Hcy, homocysteine; HDL, high-density lipoprotein; TG, triglyceride.
